# Rapid One-Step Selection Method for Generating Nucleic Acid Aptamers: Development of a DNA Aptamer against α-Bungarotoxin

**DOI:** 10.1371/journal.pone.0041702

**Published:** 2012-07-30

**Authors:** Lasse H. Lauridsen, Hadi A. Shamaileh, Stacey L. Edwards, Elena Taran, Rakesh N. Veedu

**Affiliations:** 1 School of Chemistry and Molecular Biosciences, The University of Queensland, Brisbane, Queensland, Australia; 2 The Novo Nordisk Foundation Center for Biosustainability, Technical University of Denmark, Hørsholm, Denmark; 3 Australian National Fabrication Facility, Australian Institute for Bioengineering and Nanotechnology, The University of Queensland, Brisbane, Queensland, Australia; University of Helsinki, Finland

## Abstract

**Background:**

Nucleic acids based therapeutic approaches have gained significant interest in recent years towards the development of therapeutics against many diseases. Recently, research on aptamers led to the marketing of Macugen®, an inhibitor of vascular endothelial growth factor (VEGF) for the treatment of age related macular degeneration (AMD). Aptamer technology may prove useful as a therapeutic alternative against an array of human maladies. Considering the increased interest in aptamer technology globally that rival antibody mediated therapeutic approaches, a simplified selection, possibly in one-step, technique is required for developing aptamers in limited time period.

**Principal Findings:**

Herein, we present a simple one-step selection of DNA aptamers against α-bungarotoxin. A toxin immobilized glass coverslip was subjected to nucleic acid pool binding and extensive washing followed by PCR enrichment of the selected aptamers. One round of selection successfully identified a DNA aptamer sequence with a binding affinity of 7.58 µM.

**Conclusion:**

We have demonstrated a one-step method for rapid production of nucleic acid aptamers. Although the reported binding affinity is in the low micromolar range, we believe that this could be further improved by using larger targets, increasing the stringency of selection and also by combining a capillary electrophoresis separation prior to the one-step selection. Furthermore, the method presented here is a user-friendly, cheap and an easy way of deriving an aptamer unlike the time consuming conventional SELEX-based approach. The most important application of this method is that chemically-modified nucleic acid libraries can also be used for aptamer selection as it requires only one enzymatic step. This method could equally be suitable for developing RNA aptamers.

## Introduction

In recent years, nucleic acids-based therapy has attracted significant interest for the treatment of many diseases. It comprises several approaches based on nucleic acid as the active component, which include antisense [1], [2], ribozymes [2], short interfering RNA (siRNA) [2]–[4], microRNA (miRNA) [5], [6], and aptamers [7]–[11]. Aptamer technology is one of the most promising and important approaches for therapeutic development. Aptamers are short single-stranded (ss) DNA or RNA oligonucleotides that can bind to its target with high affinity and specificity due to their ability to adopt three-dimensional shapes in solution. These structured sequence-specific ssDNA or RNA aptamers can specifically bind to a myriad of different targets [11]. Coupled with superior storage stability and easy solid-phase production steps, aptamers have become prime candidates for therapeutic and diagnostic applications. Furthermore, research on aptamers led to the marketing of Macugen®, an inhibitor of vascular endothelial growth factor (VEGF) for the treatment of age related macular degeneration (AMD) [12].

Traditionally, aptamers are generated by a process referred to as SELEX (Systematic Evolution of Ligands by Exponential enrichment) [13]–[15]. This method is very time consuming and involves several enzymatic steps. Aptamers composed of natural DNA and RNA pose some serious limitations such as poor nuclease resistance and decreased binding affinity. To combat these problems, chemically modified nucleotides are used. However, the use of modified nucleotides in SELEX is very limited because of their poor enzymatic recognition capabilities, demanding an alternative approach for aptamer selection [16], [17]. In addition, the repetitive nature of the SELEX procedure increases the risk of inducing mutations during the enzymatic amplifications steps and consequently losing important sequence information before isolating the high binding aptamers [18].

We have selected α-bungarotoxin (7984 Da) as the target in our study. α-Bungarotoxin is one of the toxic components of krait snake (*Bungarus multicinctus*) venom. The toxin binds irreversibly and competitively to the acetylcholine receptor found at the neuromuscular junction, causing paralysis, respiratory failure and ultimately death in the victim. Krait snakebite is lethal and often encountered in Southeast Asia and the Indian subcontinent. The treatment often becomes difficult unless the snake is properly spotted and also noted that there is frequently little or no pain at the site of a krait bite, which can lead to false-reassurance to the victim [19], [20].

## Results

### One-step Selection on a Glass Coverslip

**Figure 1:**
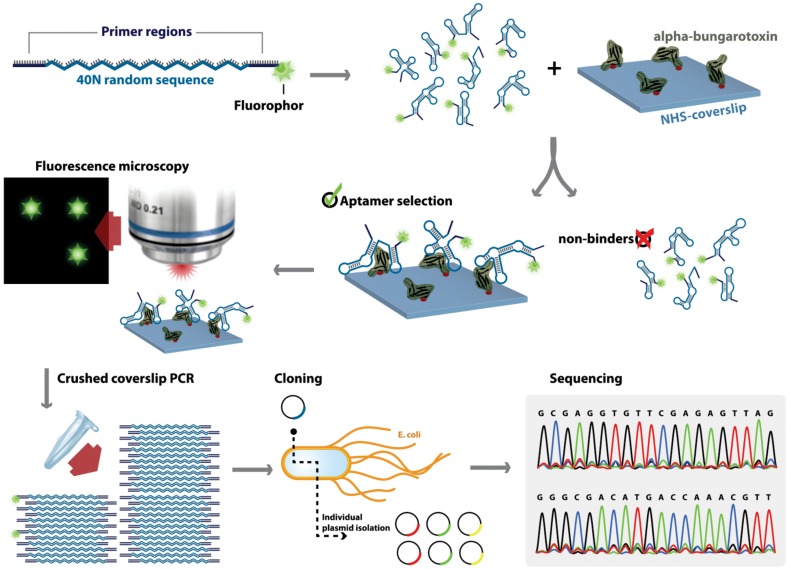
Single-step selection method. FAM-labeled oligonucleotide library containing a 40 nt random region was incubated with a target immobilized on a glass coverslip. Unbound sequences were discarded by extensive washing followed by fluorescence microscopy. The coverslip was later crushed and eluted the bound sequences by heating in water. The selected aptamers were amplified by PCR and cloned into *E.coli* and the purified individual plasmid DNA was sequenced.

**Figure 2:**
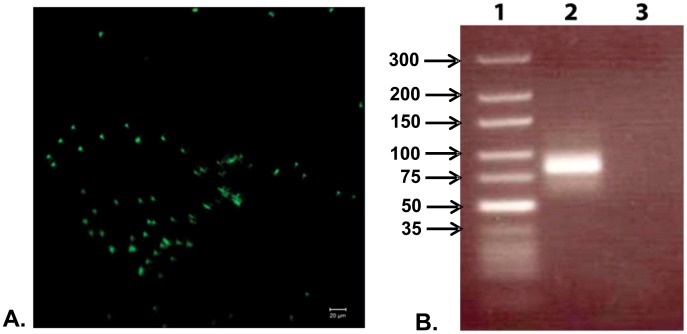
One-step selection and amplification of the aptamer candidates. A. The observed fluorescence on the toxin immobilized glass coverslip after washing. The glass coverslip was washed with extensive amounts of binding buffer, effectively removing all nonspecific adhesion to the coverslip. The pattern of highly localized fluorescent dots and the absence of background smear provide an indication of successful selection; B. PCR amplification of the eluted bound sequence. Lane 1: Marker DNA, lane 2: Amplified product from the eluted DNA aptamers, lane 3: PCR amplification without using a template DNA (negative control).

Our approach of one-step aptamer selection is schematically illustrated in Figure 1. First, the target α-bungarotoxin was covalently linked to the surface of a glass coverslip which was coated with ***N***-hydroxysuccinimide (NHS) functionalized polyethylene glycol (PEG) for reaction with primary amines of the target. The non-linked sites were blocked with a deactivation buffer supplied by the manufacturer. The coverslip is very thin and fragile that makes it difficult to work with. In our experience, it was best to handle the coverslip on a piece of Parafilm. The nucleic acid library was first incubated with a deactivated coverslip without the target peptide to remove the sequences that have affinity to the surface (negative selection). The remaining unbound pool was collected and added on to the coverslip immobilized with α-bungarotoxin and incubated overnight in a humidifying chamber. The coverslip was then washed with a large excess of the binding buffer and dried by gently blowing N_2_ gas. The aptamer binding to the target was monitored directly by fluorescence microscopy. The absence of high background fluorescence and the appearance of localized fluorescent spots indicated a successful aptamer selection against α-bungarotoxin (Figure 2A).

After the initial observation by fluorescence showing aptamer binding to the toxin, the bound aptamers were eluted by crushing the glass coverslip well and soaking in water in a 1.5 mL Eppendorf tube followed by vortexing and heating at 90°C for ten minutes. The supernatant was collected and PCR amplification yielded a single band on an agarose gel corresponding to the expected library size (Figure 2B). The purified PCR product was later cloned into *E.coli* and fifty five positive clones were collected and the purified individual plasmid DNA was sequenced. The obtained crude sequences were screened and only those sequences, which retained the initial library design with correct primer binding regions, were considered for further analyses. Although most sequences contained a particular motif -GGGC-, we have only selected the repeating sequences with the motif -GGGC- for binding studies, which are listed in Table 1.

**Table 1:** Aptamer sequences obtained from the selected clones.

Clones	Sequences
15, 22 & 42	ATCATGTCTTTTCGGGATGGGCAAGAAGGGAAATAATGC
28 &45	AGAAACGTAGCGGTAACTGCTAGAATGCGCCGAGAGAGCG
24 & 51	GCGAGGTGTTCGAGAGTTAGGGGCGACATGACCAAACGTT
1 & 16	AGGGCACAGAGAAGAAGTCGTGGATTTGAATGGTTTTGGT

Only the sequences from the random regions are shown.

### Aptamer Binding Affinity Determination

**Figure 3:**
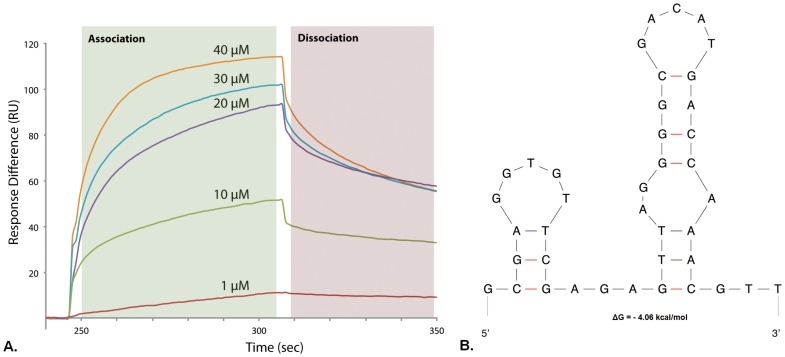
Aptamer characterization. A. Binding specificity of the aptamer obtained from clone 24 & 51 by surface plasmon resonance (SPR) technique using Biacore 3000. Various concentrations of α-bungarotoxin (1, 10, 20, 30, and 40 µM) were passed through the aptamer which was immobilized on a streptavidin coated sensor chip. The obtained sensorgrams demonstrate the aptamer binding to α-bungarotoxin; B. Predicted structure of the obtained aptamer using the DNA folding platform from mfold web server [26].

The binding interaction between the selected aptamer and α-bungarotoxin was determined by surface Plasmon resonance (SPR)-based Biacore 3000 instrument. The selected aptamer sequences were chemically synthesized which contained a biotin tag at 5′-end. The biotinylated aptamer sequences were then immobilized on a sensor chip SA (streptavidin coated chip, GE life sciences). Varying concentrations of α-bungarotoxin (500 nM –50 µM) was then passed through the surface immobilized with the aptamer. SPR data showed that the aptamer sequence of clone 24 & 51 can bind to α-bungarotoxin with a *K_D_* of 7.5 µM (Figure 3A). In contrast, aptamer sequences of clones 15, 22 & 42; 28 & 45 and 1 & 16 showed negligible binding (data not shown). We have also performed non-specific binding analyses using a non-sense target, insulin. There was no binding observed as expected, even at higher concentrations.

## Discussion

Highly sensitive and specific nucleic acid aptamers against α–bungarotoxin can be extremely useful as a diagnostic and therapeutic against krait snake envenomation. Aptamers offer a unique alternative to the existing antibodies against the toxins which are much larger, easily degraded, expensive and require the use of animals for a laborious time consuming production [9], [21]. On the other hand, aptamers are easily synthesized and can be modified with reporter molecules or other nucleotide analogues to enhance their properties like nuclease resistance. Aptamers also adopt unique sequence-dependent three-dimensional shapes, so they are in a sense, shape libraries that may bind to a target based on their sequence, conformation and/or charge through interactions with binding pockets, hydrogen bonds, stacking of aromatic rings, van der Waals forces, or a combination of these [21]. Aptamers are normally developed by SELEX, an *in vitro* evolution process developed around twenty years ago. Since the invention, several SELEX protocols employing different selection approaches have been developed [13], [22]. The majority of these modified protocols still require repetitive selection and enrichment steps for selecting high affinity aptamers. Considering the increased interest in aptamer technology globally that rival antibody approach, a simplified selection, possibly in one-step, technique is required for developing aptamers in limited time period and to avoid repeated enzymatic steps that might induce sequence mutations. In this direction, there are few reports showing the selection of aptamers in a single step [23]–[25]. One approach was called MonoLEX in which a DNA aptamer was selected against the Vaccinia virus [23]. The technique involved an affinity chromatography step followed by subsequent physical partitioning of the affinity resin and PCR amplification of the bound aptamers. Another method to develop aptamers in one step was by using a combination of atomic force and fluorescence microscopy called NanoSelection [24]. However, the major problem with this approach was that the selection was carried out with a very small pool of randomized preselected thrombin binding aptamer oligonucleotides unlike a conventional SELEX library and there was no further report on aptamer selection against new targets by this approach. Krylov and co-workers reported a Non-SELEX selection of aptamers using capillary electrophoresis by which they were able to develop aptamer with affinity in the low micro molar range [26].

We have developed a one-step method for rapid selection of aptamers that is simple, cheap and user-friendly. For successful aptamer selection, three key steps that need to function optimally; 1) efficient separation of the target bound aptamers, 2) enzymatic amplification by PCR and 3) regeneration of the selected single-stranded aptamers. One major highlight of our method is that it eliminates the need for repeated enzymatic amplification and regeneration of the single-stranded aptamer that always complicate and affect the selection success. In one step selection method, we have adopted an easy to use glass coverslip for immobilizing the target for selecting aptamers by monitoring fluorescence under a microscope to ensure that we are in fact selecting and processing the target bound aptamer. One-step selection of aptamers on a glass coverslip is an easy way for generating aptamers even for a small biomolecular target like α-bungarotoxin (7984 Da) used in this study. The target immobilization on to the coverslip was verified by using an Alexa Fluor 555 dye-labeled α-bungarotoxin followed by fluorescence microscopy (Figure S1B). A large pool of fluorescein (FAM) dye–labeled DNA oligonucleotides (100 µL of 40 µM, contains 2.4×10^15^ members, the protocol allows using much larger sized libraries, however, this may increase the background fluorescence and force non-specific binding) was then incubated with the target-immobilized coverslip. We speculate that a stringent washing procedure employed might also account to a great extent for the successful aptamer identification by this approach followed by fluorescence microscopy. The coverslip was later viewed under a microscope and observed the green spots indicating that we have selected an aptamer. A control experiment was performed to make sure that we have selected the aptamers against the intended target peptide immobilized on to the coverslip. For this purpose, we used the Alexa Flour 555-labeled α-bungarotoxin and FAM-labeled DNA library and performed the selection as mentioned. Later, we did overlay the images. A target specific selection should turn to an orangish-yellow color upon overlay. If not, both the target and the aptamer will remain as red and green spots respectively. In our case, we have seen very distinct orangish-yellow spots upon overlay reassuring the target specific aptamer selection (Figure S1). Based on this experiment, we believe that the aptamer was indeed selected specifically to α-bungarotoxin.

Negative selection using a deactivated coverslip is also a necessary step prior to the actual aptamer selection against the target. However, it is important to mention that some of the most potent binders to α-bungarotoxin that might also have affinity for the coverslip surface will be removed by this step even before the actual aptamer selection. But, in this selection protocol this negative selection is required mainly to avoid the non-specific surface binding aptamers. Majority of the glass surface binding aptamers can be removed by this step and other week non-specific binders can subsequently be washed away by very stringent washing procedure. To further verify this, we have performed one selection without performing negative selection using a deactivated glass coverslip. But in this case, we observed an increased amount of green fluorescence on the coverslip indicating nonspecific selection of the aptamers against the glass surface (data not shown). The binding affinity (*K*_D_ = 7.52 µM) of the identified aptamer (from clone 24 & 51, Table 1) against α-bungarotoxin is not very high. The binding affinity can be improved by truncating the selected aptamer based on its predicted structure by mfold web server (Figure 3B) [27]. Although we cannot generalize, we believe that using our method, the binding affinity can be improved by adopting various strategies. Using a larger target protein (>15 kD) may improve the probability in selecting high affinity aptamers to some extent because of their structural diversity and immobilization efficiency on the coverslip. We also foresee that by combining capillary electrophoresis [26], [28] and our one-step protocol may yield high affinity aptamers, ie, the target bound aptamer candidates are first separated by capillary electrophoresis followed by incubation on the target immobilized coverslip to remove all week binders monitored by fluorescence microscopy. It is noteworthy to mention that our selection protocol allows aptamer selection within 24 hrs, much shorter than a month long conventional SELEX selection procedure.

In summary, we have demonstrated a simple, rapid one-step selection protocol for developing nucleic acid aptamers. Using this method, one DNA aptamer against α-bungarotoxin was identified with a dissociation constant of 7.5 µM. This method opens up new ways for aptamer selection in a very limited time at low cost. So far, the use of important chemically-modified nucleotides in aptamer selection is limited due to their poor enzymatic recognition capabilities. Our approach may prove useful especially for using modified nucleotides containing library-based aptamer selection. Although we reported a DNA aptamer selection using this approach, it could equally be suitable for developing RNA aptamers.

## Materials and Methods

All DNA oligonucleotide sequences were purchased from Integrated DNA technologies (Coralville, USA). The *N*-hydroxysuccinimide activated glass coverslip was purchased from MicroSurfaces Inc (supplied by Stratech Scientific, Australia). Phusion DNA polymerase was purchased from New England Biolabs (supplied by Genesearch Australia). NucleoSpin® Extract II kit for PCR clean up was purchased from Macherey-Nagel (supplied by Scientifix Australia). For cloning the PCR product, the plasmid pCR®-Blunt was purchased from Invitrogen, Australia. Plasmid DNA was extracted and purified using a QIAprep Miniprep Kit (purchased from Qiagen, Australia). Fluorescence microscopy was performed using a Laser Scanning Microscope (LSM) 710 from Zeiss and a 20x objective at an excitation wavelength of 488 nm and laser power of 1 mW.

The designed nucleic acid library contained a 40nt random region flanked by two primer binding regions (5′-GGACAGGACCACACCCAGCG-40nt-GGCTCCTGTGTGTCGCTTTGT-FAM-3′) and the library was labeled with a fluorescent tag (FAM) and purchased from IDT in 1 µmol scale. The library was dissolved in 1×PBS containing 5 mM MgCl_2_ to yield a concentration of 40 µM and denatured by heating at 90°C for 5 min followed by cooling in ice. Before use, the library was kept at room temperature for 15 min to adjust the temperature.

### Single-step Selection on Glass Coverslip

The *N*-hydroxysuccinimide activated glass coverslip was treated as per the protocol recommended by the manufacturer. In short, 25 µL of the α-bungarotoxin (125 µM) was dissolved in 30 µL PBS containing 10% glycerol and applied drop by drop on to the NHS activated coverslip which was placed on a piece of Parafilm® M and incubated for 30–40 min at 24°C in a humidifying chamber. The glass coverslip was then washed with 3×200 µL washing buffer (1×PBS containing 0.05% Tween 20) by adding droplets to a piece of Parafilm® M and then by gently placing the coverslip upside down soaking the coverslip in 200 µL washing buffer and waiting for 5 min at 24°C. The coverslip was then placed in 300 µL deactivation buffer (supplied by the manufacturer) to block any unreacted groups for 35 minutes in a humidifying chamber. The coverslip were subsequently washed two times with PBS containing 0.05% Tween 20. After this immobilization step, 120 µL of the prepared library was incubated for 30 min on an inactivated coverslip without α-bungarotoxin immobilization (negative selection). Then, 100 µL of the counter-selected nucleic acid library was transferred to the α-bungarotoxin immobilized coverslip and incubated overnight at 24°C in a humidifying chamber. Extensive washing was then performed with 4 mL washing buffer (5×800 µL), followed by drying the coverslip by gently blowing nitrogen gas. The backside of the coverslip was cleaned with a clean tissue soaked in ethanol before fluorescent imaging by microscopy.

The toxin bound aptamers were later eluted by crushing the glass coverslip in an Eppendorf tube followed by heating in Milli-Q water at 90°C for 20 minutes. The resulting solution was centrifuged at 14000 rpm and the supernatant was collected for the subsequent PCR amplification step. In short, the PCR mixture was prepared in a total volume of 50 µL by adding 10 µL 5× Phusion HF buffer (included in the Phusion DNA polymerase kit), 4 µL of dNTPs (400 µM), 28 µL of two times distilled water, 1.5 µL of forward primer (50 µM), 1.5 µL of reverse primer (50 µM), 5 µL of template (supernatant) and 0.5 µL of Phusion DNA polymerase (250 U/µL). The reaction mixtures were gently vortexed and then amplified using a thermal cycler (S1000™ Thermal cycler, Bio-Rad). A 25-cycle PCR consisted of denaturation at 98°C for 10 seconds, annealing at 55°C for 15 seconds and extension at 72°C for 25 seconds. After the polymerase reactions, gel-loading buffer (included in Ultra Low DNA size marker kit from Fermentas, supplied by VWR Australia) was added (1.5 µL) and the products were analysed by 4% agarose gel electrophoresis followed by UV-photography. The PCR product was later purified by using the NucleoSpin® Extract II kit.

### Cloning and Sequencing

Purified PCR products (50 ng) were cloned into *E.coli* using a pCR®-Blunt (Invitrogen) vector according to the manufacturer’s instructions. Plasmid DNA was extracted using QIAprep (Qiagen) and sequenced by the Australian Genome Research Facility (AGRF, Brisbane, Australia).

### *K*_D_ Determination by SPR

The binding affinities of the selected aptamers against α-bungarotoxin were analyzed by Surface Plasmon Resonance using a Biacore 3000 Instrument (GE) at 24°C. Streptavidin immobilized sensor chip SA (GE, for Biacore 3000) was used for all measurements of kinetics and 1×PBS binding buffer (137 mM NaCl, 2.7 mM KCl, 4.2 mM Na_2_HPO_4_, 1.47 mM KH_2_PO_4_, 0.005% Tween 20) was used as the running buffer. The sensor chip was preconditioned with 1 mL of the running buffer at a flow rate of 100 µL/min. Then, the 5′-biotinylated aptamer sequences were immobilized on the chip as recommended by manufacturer’s manual. Varying concentrations of α-bungarotoxin was then injected at a flow rate of 30 µL/min for 2 min. The surface was regenerated by treating with 10 mM NaOH. The *K*_D_ values were calculated on the basis of a 1∶1 Langmuir binding model by fitting the association and dissociation rates using the Biacore 3000 evaluation software.

## Supporting Information

Figure S1 **Fluorescence-based characterization of the α-bungarotoxin aptamer selection by one-step protocol.** A. An overlay image (orangish-yellow spot indicating aptamer binding to α-bungarotoxin) of the immobilized α-bungarotoxin labeled with Alexa Fluor 555 (red colour) and the toxin bound FAM-labeled DNA aptamer (green fluorescence); B. Characterization of the α-bungarotoxin labeled with Alexa Fluor 555 (red colour) immobilized on a glass coverslip; C. α-bungarotoxin bound FAM-labeled DNA aptamer (green fluorescence).
(TIF)
